# Orchitis Due to Mumps without Salivary Gland Swelling

**Published:** 1910-02-12

**Authors:** 


### Medicine.

### ORCHITIS DUE TO MUMPS WITHOUT SALIVARY GLAND SWELLING.

The importance of remembering that orchitis may be due to mumps is considerable, particularly in young men in whom otherwise the gonoccoccus may sometimes be put down too readily as the cause of the mischief. Mumps is a general, specific, infectious disease comparable to the exanthemata, and its commonest local manifestation is the painful swelling of various glands. So constantly are the salivary glands, particularly the parotid glands, inflamed and enlarged that one is a little apt to forget that mumps may occur without any salivary gland affection at all, and that painful swelling of the testes may be its only manifestation in young adolescent males.

The chronological relation between the parotid and the testicular manifestations of the disease is variable. Parotitis usually precedes orchitis by one or more days; exceptionally parotid inflammation follows orchitis instead of preceding it; and there are rarer cases still in which tumefaction of the salivary glands is entirely absent, and orchitis may then be the sole manifestation of the disease.

The following is an instance in point. An acute orchitis occurred in a boy of 15; the cause of the orchitis was not obvious, but the nature of the case was cleared up by the development in each of the patient's two sisters, 15 days and 23 days later respectively, of the typical parotid inflammation of mumps. The boy in question was seized suddenly on April 20 with a violent headache and repeated vomiting. When seen by his doctor shortly afterwards, so little could be found to account for his symptoms that no better diagnosis than gastric fever could be suggested. From April 20 to April 23 his condition remained about the same, except that a purgative had been followed by a return of appetite and a diminution of the fever. On April 24, four days after the onset of the illness, the lad on rising in the morning felt that his right testicle was slightly painful. The next day—April 25—it had become very painful and it had swollen, and the skin over it was red and œdematous, so that the patient was obliged to stay in bed.

He was very carefully examined by a surgeon, who, finding that the epididymis was even more affected than the testes, and that nowhere, either in the urethra or elsewhere, was there any obvious cause for this trouble, debated whether it might not be an example of acute tuberculous epididymo-orchitis. At this time the temperature had risen to 104° F., which was the highest it had yet attained.

On April 27 a more careful inquiry into the history elicited the fact that on April 10, which was the last day the boy had been at school before the break-up for the holidays, and 10 days before the onset of the illness, three schoolmates had been laid up with mumps. This seemed to throw considerable light upon the case. Under treatment by means of rest and fomentations the orchitis rapidly decreased and it was perfectly cured by May 2. If there had been the least doubt as to the orchitis in this case being due to mumps, this doubt was entirely removed on May 5, that is to say, 15 days after the boy had been taken ill, when his elder sister, aged 16, developed great swelling of the right parotid gland which was very painful, the swelling next day having also extended to the left. Her condition was undoubtedly that of mumps, which she in all probability caught from her brother. A younger sister, aged 11, also developed the typical symptoms of mumps on May 13.

